# Locomotion as a Powerful Model to Study Integrative Physiology: Efficiency, Economy, and Power Relationship

**DOI:** 10.3389/fphys.2018.01789

**Published:** 2018-12-11

**Authors:** Leonardo Alexandre Peyré-Tartaruga, Marcelo Coertjens

**Affiliations:** ^1^Exercise Research Laboratory, Universidade Federal do Rio Grande do Sul, Porto Alegre, Brazil; ^2^Postgraduate Program in Pneumological Sciences, Hospital de Clínicas de Porto Alegre, Porto Alegre, Brazil; ^3^School of Physical Therapy, Federal University of Piauì, Parnaìba, Brazil

**Keywords:** efficiency, economy, metabolic cost, mechanical work, self-selected walking speed, optimal walking speed, gait

## Abstract

Locomotion is the most common form of movement in nature. Its study allows analysis of interactions between muscle functions (motor) and lever system arrangements (transmission), thereby facilitating performance analysis of various body organs and systems. Thus, it is a powerful model to study various aspects of integrative physiology. The results of this model can be applied in understanding body functions and design principles as performance outputs of interest for medical and biological sciences. The overall efficiency (*eff_overall_*) during locomotion is an example of an integrative parameter, which results from the ratio between mechanical output and metabolic input. Although the concepts of cost (i.e., metabolic expenditure relative to distance) and power (i.e., metabolic expenditure relative to time) are included in its calculation, the *eff_overall_* establishes peculiar relations with these variables. For a better approach to these aspects, in this study, we presented the physical-mathematical formulation of efficiency, as well as its conceptual definitions and applications. Furthermore, the concepts of efficiency, cost, and power are discussed from the biological and medical perspectives. Terrestrial locomotion is a powerful model to study integrative physiology in humans, because by analyzing the mechanical and metabolic determinants, we may verify the efficiency and economy relationship through locomotion type, and its characteristics and restrictions. Thus, it is possible to elaborate further on various improved intervention strategies, such as physical training, competition strategies, and ergogenic supplementation.

## Introduction

Locomotion, i.e., displacement through an environment, is one of the most significant behaviors in the Animalia kingdom. As evolutionary pressures impose specific adaptations in the forms and functions of animals, natural locomotion is a type of movement that is readily sensible to these pressures (Dickinson et al., 2000). Adjustments resulting from environmental constraints (such as environment, gradient, terrain, and temperature), tasks (such as type of locomotion, load, and speed), and individual characteristics (such as age, sex, locomotor restrictions, and disease) justify locomotion as a model to test the functions of a locomotor apparatus, such as cardiorespiratory or metabolic system, through the analysis of integrative parameters, such as efficiency and economy.

The term “efficiency” has a broad application in the fields of economy, engineering, biology, and politics. The origin of this term is antique, and philosophers, such as Hobbes and Bacon, used it to denote anything that caused a consequence. This definition is currently used in two ways: value-neutral qualitative (by description) or quantitative (by measurement) characterization of processes, machines, or practices (for general definitions, see Schipper, 1998). As discussed ahead, the term “qualitative” is often used to denote (and sometimes confused with) something improved or enhanced. Its use became quantitative with the advent of the industrial revolution. Gilbert defined it in his lecture as the president of the Royal Society of London, in 1827: “The criterion of their (ordinary machines) efficiency is force (f) multiplied by the space (s) through which it acts (f × s); and the effect which they produce, measured in the same way, has been denominated duty…” (Gilbert, 1827). Recently, Blake in his milestone book (Blake, 1991), proposed to define efficiency from an evolutionary perspective as a measure of performance relative to a physical or biophysical process or law. Moreover, using one of the main examples, we will apply the term efficiency (*eff*) in this study to characterize the fraction of the amount of metabolic energy (criterion; input) that can be transformed into mechanical work (duty; output). Interestingly, some combinations of counterparts are observed, thereby providing a robust experimental model for investigations of the integrative physiology of systems. Applications of *eff* are useful in areas of organismic and comparative biology, movement disorders, cardiorespiratory diseases, and physical/sports performance. The analysis of metabolic energy according to the measurable parameters in the whole body and the fluctuations of mechanical energy output of the internal and external counterparts are analyzed as follows.

The efficiency establishes a relationship with economy and power through the denominator (metabolic input) that can be calculated as *P*_metab_ (the energy expenditure as a function of time, *P*_metab_) or metabolic cost (the energy expenditure as a function of distance traveled, *C*_metab_). The *C*_metab_ parameter of walking in humans and many cursorial animals, as a function of speed, can be represented by a U shape, with the lowest point at the so-called OWS that corresponds to the SSWS. This phenomenon is explained by the pendular mechanism, in which the highest pendular recovery coincides with the lowest value of *C*_metab_ (Cavagna and Kaneko, 1977; Gomeñuka et al., 2016). In some pathological conditions, such as chronic heart failure, Parkinson disease, chronic obstructive pulmonary disease, and amputees, humans choose to walk at speeds below their OWS. This deviation from the normal condition has repercussions on locomotor energy and performance. Further, understanding the differences between *C*_metab_ and *P*_metab_ might be useful in unveiling aspects applied to aerobic exercise prescription for normal and pathological individuals.

The numerator of efficiency is expressed as *C*_mec_ or *P*_mec_, and represents the minimum muscle-tendon work performed to maintain the movement. The mechanical work by definition can be segregated into internal (Wint, energy fluctuations of segments with respect to the center of body mass) and external (Wext, energy fluctuations of the center of body mass with respect to the external environment or surroundings) work (Cavagna and Kaneko, 1977). One clear application of mechanical work and efficiency in locomotion studies was assessed in a pioneer study by Margaria (1938), which stated that at extreme downhill and uphill slopes, the efficiency is similar to the efficiency of negative and positive muscular work, respectively. In bouncing gaits on a level ground, the mechanical counterpart increases at levels higher than *P*_metab_, indicating a contribution of elastic mechanism in these gaits (Cavagna et al., 1964).

Considering the new techniques in biomechanics and physiology, such approaches might help uncover novel interpretations and regulators of body functions in an integrative manner. The first objective of this study is to describe concepts, such as efficiency, *P*_metab_, and *C*_metab_, applied to locomotion. The second objective is to analyze the limitations and possibilities of efficiency caused by different constraints, such as aging, physical fatigue, and movement disorders. In this study, we will not analyze any cellular and molecular phenomena. However, central and peripheral restrictions will be included in the integrative analyses of muscle, which will be considered as “motor,” as well as the corresponding interaction with the “machine” (the skeletal lever system).

### Physical-Mathematical Definition of Efficiency

The definition of efficiency applied to locomotion is well discussed by Cavagna (2010, 2017). In this study, we will summarize the proposition. During movement, the first function of a muscle is to produce positive mechanical work (W+, shortening). The energy capable of supporting this work is produced by hydrolysis of adenosine-tri-phosphate inside the muscle and denominated fuel (*C*_metab_ and/or *P*_metab_).

The *eff_musc_* including the biological “motor” (muscles) is represented as follows:

(1) $$eff_{musc}=W+/fuel$$

As discussed earlier, the energetic efficiency might be analyzed in many physiological processes. The efficiency at which an animal can use the metabolic energy contained in the food is essential information for nutritionists and ecologists (Webster, 1981). We also have the molecular efficiency feature, e.g., the muscle fiber type. The analysis of molecular composition and contractile properties of minute portions of skinned muscle fiber has facilitated investigation of functional and molecular diversity of skeletal muscle fibers (He et al., 2000). All these concepts have a strict relationship with the movement performance of an animal according to the functional or biomechanical demands.

In terrestrial locomotion, we use the appendages that are responsible for the inefficiency of legged locomotion (Cavagna, 1978). The value of *C*_metab_ in terrestrial locomotion is relatively higher in comparison to that in aquatic and aerial locomotion (Schmidt-Nielsen, 1972), although the density of air is 800 times lower than water (Di Prampero, 1986). The collisional characteristic of terrestrial locomotion because of the ground support induces huge fluctuations in the mechanical energy of a body (total mechanical work, Wtot). Thus, we have the second efficiency, which is regarding the machinery (the musculoskeletal lever system), characterized as the fraction of W+ that is transmitted to Wtot, and denominated as transmission efficiency (*eff_transmission_*, Equation 2):

(2) $$eff_{transmission}=Wtot/W+$$

The *eff_overall_* is the product of these two efficiencies (Equation 3).

(3) $$eff_{overall}=eff_{musc}.eff_{transmission}$$

Further, substituting Equations 1 and 2 in Equation 3, we have (Equation 4)

(4) $$eff_{overall}=(W+/fuel).(Wtot/W+)$$

Furthermore, canceling W+ (Equation 5)

(5) $$eff_{overall}=Wtot/fuel$$

As discussed ahead, *eff_overall_* corresponds to a broader and more complex expression of the *eff_mec_*. By convention and practicality, *eff_overall_* will be expressed as *eff* as well in this study.

The *eff_musc_* parameter is constituted by two more efficiencies: efficiencies of phosphorylative coupling (*eff_p_*) and contraction coupling (*eff_c_*). The efficiency of phosphorylative coupling refers to the phosphorylation rate (high-energy inorganic phosphates production) obtained from oxidation of energetic substrates through aerobic metabolism (P:O ratio) and from caloric equivalent of adenosine-tri-phosphate. The efficiency of contraction coupling is the ratio of the produced tension during muscle contraction and the quantity of released energy from high-energy phosphates. The *eff_musc_* is obtained through the product of both efficiencies (around 60 and 49%, respectively), which results in 25–30% (Whipp and Wasserman, 1969; Woledge et al., 1985).

### Conceptual Definitions and Methodological Approaches for Efficiency

Considering various methodological reasons, the study of human and animal energies during locomotion activities allows the use of different expressions for the term efficiency. Further, gross efficiency refers to the ratio between *P*_mec_ produced and total *P*_metab_ expended during the activity, including energy expenditures related to the functioning of organs necessary for maintenance of life. The term net efficiency subtracts the energy expenditure assessed in the resting situation from the denominator, i.e., it refers only to *P*_metab_ consumed during the activity (Cavanagh and Kram, 1985b). The denomination “work efficiency” is used in subtracting *P*_metab_ expended to perform the work of moving the lower or upper limbs (Wint) on a cycle ergometer (Whipp and Wasserman, 1969), expressing efficiency only for Wext production. Delta efficiency refers to the efficiency of *P*_mec_ variation (load or speed) over *P*_metab_ variation during exercise, i.e., it is an efficiency related to range of intensities (Gaesser and Brooks, 1975; Donovan and Brooks, 1977). Instantaneous efficiency have similar characteristics to delta efficiency; however, it refers to an infinitesimal decrease of *P*_mec_ and *P*_metab_ variations (Stainbsy et al., 1980). Some authors have used the expression “apparent efficiency” to express the influence of energy-saving mechanisms in the calculation of efficiency (Åstrand, 2003; Minetti et al., 1999). The production of mechanical work performed by the release of elastic energy stored during the stretching of muscle-tendon structures (Alexander, 1989) is one such example.

The different ways of naming an efficiency express not only methodological distinctions but also different perspectives regarding the understanding of the efficiency. These differences sometimes cause misunderstandings about the term’s usage, and excellent reviews have been produced to discuss this topic in detail (Stainbsy et al., 1980; Cavanagh and Kram, 1985b). For instance, the terms *eff_musc_* and muscular efficiency are quite similar and have similar maximal values; thus, both are considered as synonyms. Moreover, muscular efficiency has been associated with performing full-body activities. The question is that some full-body activities have efficiencies above 25–30% (Cavagna and Kaneko, 1977; Minetti et al., 1999). In these situations, the term muscular efficiency differs from *eff_musc_* (Woledge et al., 1985), thereby generating conceptual misunderstandings.

Criticisms of these findings with values above 25–30% are related to the exclusion of resting metabolism (net efficiency), which would overestimate the efficiency values (Stainbsy et al., 1980) and the way of understanding and assessing the mechanical work. In the first case, Schmidt-Nielsen (1972) suggests that for studies that need to estimate the total energy expenditure, the resting metabolism value should not be subtracted (gross efficiency), whereas for those in which efficiency is explicitly related to exercise, subtraction should be performed (net efficiency). In second case, activities in which mechanical work is generated through the release of elastic energy stored in the muscle-tendon structures, the mechanical work production may be higher or may need less metabolic energy in a relative way, thereby increasing the efficiency. In addition, transduction between the potential and kinetic mechanical energies of the center of body mass (called the “inverted pendulum” mechanism) allows part of the Wv against gravity (Wv) and forward (Wf) to be no longer produced by muscle contraction, thereby saving metabolic energy (Cavagna et al., 1976; Willems et al., 1995). When the pendulum mechanism is considered, the evaluated efficiency tends to be higher than the *eff_musc_*. Another theoretical-methodological point is the evaluation of Wint, an addition to Wtot that results in increased efficiency. Therefore, when these mechanical energy exchanges are not considered or when the resting metabolism value is not subtracted, the whole-body efficiency can be similar to the *eff_musc_* (Whipp and Wasserman, 1969; Donovan and Brooks, 1977).

Non-inclusion of mechanical work performed during the negative phase of displacement of the center of body mass (W-) to calculate total work (Wtot = Wext + Wint) is another source to discuss the differences with *eff_musc_*. In activities on a level/flat terrain, the amount of W- is equal to W+. Further, the efficiency of W- is close to 1 (or 100%); thus, the value of *C*_metab_ is very low. Therefore, only W+ is usually employed in efficiency evaluation during walking and running on a flat terrain (Willems et al., 1995). The efficiency of locomotion is frequently assessed only as the efficiency of positive mechanical work (*eff*+). However, valuable information has been obtained by analyzing W- separately during activities performed at negative slopes (Minetti et al., 1993, 1994a; Dewolf et al., 2017) and with rapid change of direction speed (Zamparo et al., 2016; Minetti and Pavei, 2018); and calculating the *eff*-. Both contractions have different efficiency values. W+ denotes the work performed to raise and accelerate the center of body mass utilizing concentric contractions, whereas W- denotes the work performed to decelerate and reduce the height of the center of body mass employing eccentric contractions. Another important aspect is that W- causes more repercussions in the calculation of Wext than those in the calculation of Wint. In these cases, the efficiency of the W-, which influences Wext directly, is considered. Thus, to control the effect of Wel from the “apparent efficiency” calculation, subtraction needs to be performed only from Wext (Minetti et al., 1994a). Furthermore, Wel is only a component of W- (Minetti et al., 1994a).

Efficiency can be expressed in various other ways, such as the term *eff_mec_*. Although initially employed in the investigation of the isolated muscle (Hill, 1913), studies related to *eff_mec_* were later extended to full-body research (Hill, 1922). Currently, the usage of this marker has the advantage of expressing and including Wtot (=Wext + Wint) produced by a body during an activity (Willems et al., 1995; Saibene and Minetti, 2003). This expression is criticized because it is not thermodynamically compatible with the characteristics of *eff_musc_* (Whipp and Wasserman, 1969), as initially proposed by Hill. The *eff_mec_* parameter is considered to be an inappropriate concept from the thermodynamics perspective (Whipp and Wasserman, 1969). This argument is attributed to the fact that Wtot is usually determined in locomotion, which includes the muscular work summed to the elastic Wext, without the corresponding metabolic increment. These situations would not be in agreement with Gibbs free energy, which refers to the portion of the ΔH expected to produce work. Accordingly, the work resulting from Gibbs free energy would be adequately assessed through work efficiency (Whipp and Wasserman, 1969) corresponding to the mechanical work without energy-saving mechanisms. Therefore, work efficiency represents the most essential of the efficiencies evaluated during exercise with the predominance of concentric contractions and without the energy-saving mechanisms (Whipp and Wasserman, 1969). The result of work efficiency is similar to the product of the *eff_p_* e *eff_c_*. Thus, the values of work efficiency are also compatible with muscle and muscular efficiencies, because of subtraction of *P*_metab_ from the measured movement of the limbs.

In recent years, the use of the term *eff_mec_* has become more complex because of the inclusion of work produced by a body under different constraints (such as friction, drag, and gravity), in different environmental conditions (such as different soil types, fluid resistance, and planets), and with or without different types of accessories and/or equipment (such as skis, fins, swim paddles, shoes, roller skates, boards, poles, and bicycles). As discussed previously, *eff_mec_* has been analyzed more broadly through *eff_overall_*, including energetic effects of equipment and accessories used during locomotion (Zamparo et al., 2002; Minetti, 2004, 2011).

Besides, the concept of efficiency is sometimes confused with general performance. However, the performance or task outcome (race time, functional test result) is related to the term effectiveness (Full, 1991). The reason for this misconception is because efficiency is understood as the inverse of the economy or *C*_metab_, i.e., as the amount of metabolic energy consumed to perform a determined task. Thus, its association with performance is inevitable, because efficiency and economy may have a deterministic relationship (as observed in section “Physical-Mathematical Definition of Efficiency”) and economy is considered an important parameter for determining the physical performance (Williams and Cavanagh, 1987). Although the two concepts denote the similar integrative energetic phenomena, the premise of a direct determination between them is not strict for all situations, as discussed in Section “Two Approaches for Exploring Efficiency and Economy Relationship in Terrestrial Locomotion.” Accordingly, the various methods used to conceptualize and understand the term efficiency has influenced the evaluation and interpretation of data, causing conflicting results. Therefore, in this study, efficiency is not synonymous with economy, and economy will be considered here as the reciprocal of *C*_metab_, as observed firstly by Margaria (1938), and recently demonstrated by Pontzer (2017).

## Efficiency, Economy, and Power Relationship

### Two Approaches for Exploring Efficiency and Economy Relationship in Terrestrial Locomotion

Currently, efficiency is related to the concept of energy saving. For instance, a machine or any efficient electronic device should necessarily have low energy expenditure, cost, or consumption (economy). This notion extends to more complex contexts, such as an industry or an institution, or even a country. This concept of efficiency has also been used in the analysis of energetics repercussions during locomotion, when the efficiency and *C*_metab_ present a strictly inverse relationship (Åstrand, 2003; Plowman and Smith, 2011).

However, this is not the case always. Although related, the concepts of economy and efficiency are different. For example, studies show that increases in energy efficiency in the United States during the 1990s came along a per capita increase in energy consumption and carbon emissions (Moezzi, 2000). Therefore, the predominant understanding of efficiency may cause misinterpretations when applied to different phenomena by establishing a deterministic relationship with the economy (i.e., higher efficiency, greater economy). Thus, the question is to what extent is it possible to apply this rationale? Or, whether it is possible to extend this approach to different conditions, including when it comes to locomotion energetics. The mode that relates efficiency to economy and *vice versa* may generate different scientific interpretations.

**FIGURE 1:**
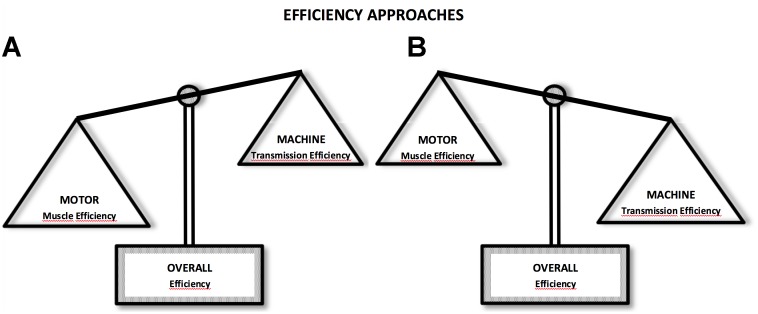
Efficiency approaches: in the first **(A)**, muscle efficiency (motor) characteristics are more predominant during locomotor activity, influencing efficiency (overall) responses; in the second **(B)**, transmission efficiency (machine) characteristics are predominant. In these cases, overall efficiency response may be higher and with different behavior than muscle efficiency. Lower values can be seen during situations under effect of isometric contractions and muscle coactivation.

The relationship between economy and *eff* during locomotion is not necessarily deterministic. Therefore, we will approach this question from two perspectives: first, an inverse relationship with both *eff* and *C*_metab_ present a quasi-parabolic behavior in phase opposition, i.e., the speed or intensity at which *eff* is observed coincide with the intensity at which maximal economy values are observed (*eff* = 1/*C*_metab_) and maximal *eff* values, are around 25–30% (Figure 1A). This result is commonly observed during the analysis of *eff_musc_* in isolated muscles at different speeds of concentric contractions (Hill, 1922, 1964; Woledge et al., 1985). Moreover, in the second approach, the *eff* and *C*_metab_ do not establish an inverse relationship, sometimes do not present quasi-parabolic behavior and, when the *eff* and *C*_metab_ show the behavior, these is not in phase opposition (*eff* ≠ 1/*C*_metab_). Further, the maximal *eff* values may be higher than 25–30%, which is different from the values of *eff_musc_* (Figure 1B).

**FIGURE 2:**
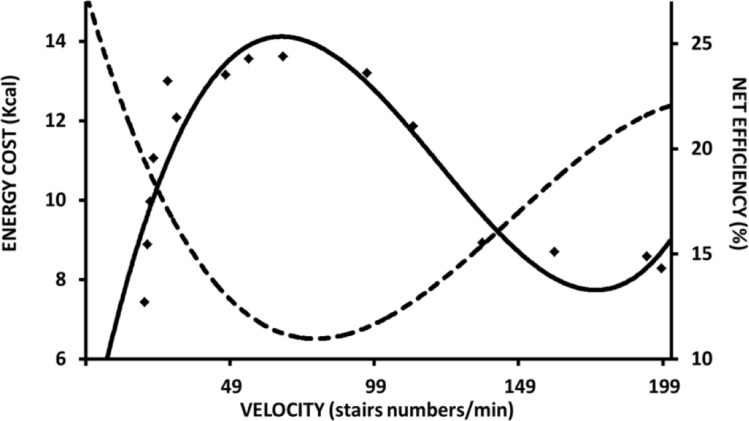
Metabolic energy cost and overall efficiency during stair climbing test at different velocities. Data and figure adapted from Lupton and Hill (1923).

**FIGURE 3:**
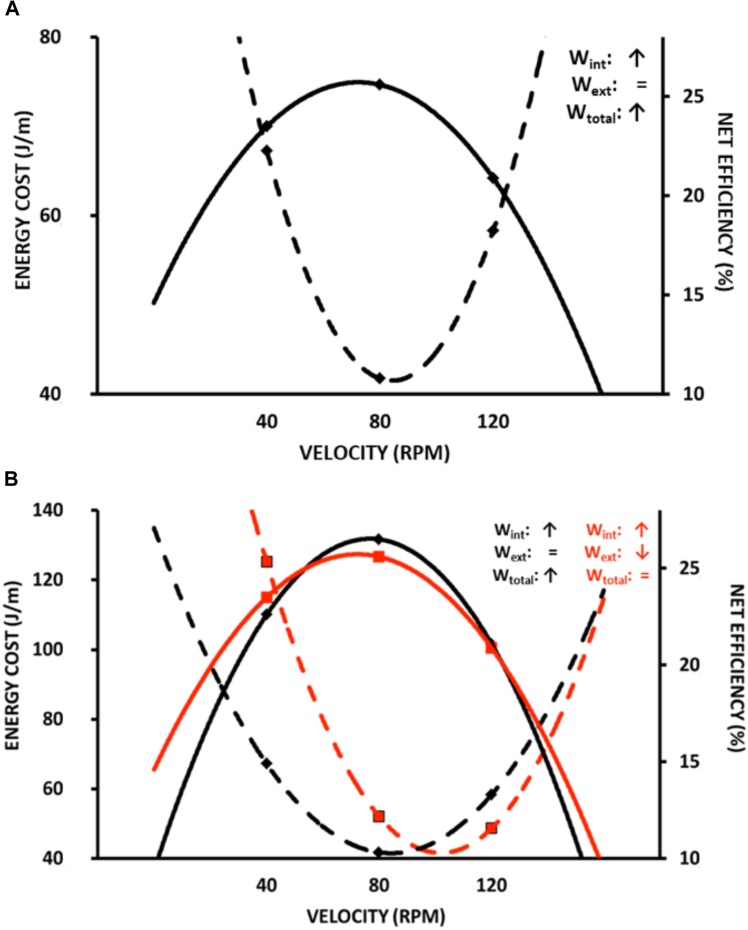
Metabolic energy cost and overall efficiency during cycling ergometer test at different velocities (revolutions per minute, RPM). **(A)** overall efficiency-economy behavior similar at muscle efficiency-economy relationship (in this case, Wtot increases because of increased Wint, while Wext is similar); **(B)** comparing energy cost and overall efficiency between different modes of work calculation: black lines are the same of **(A)**; red lines refers to a modification at work (Wint, Wext and Wtot) in relation to the black lines. Wint corresponding cycle ergometer velocity and Wext cycle ergometer load. Data adapted from Tokui and Hirakoba (2007).

**FIGURE 4:**
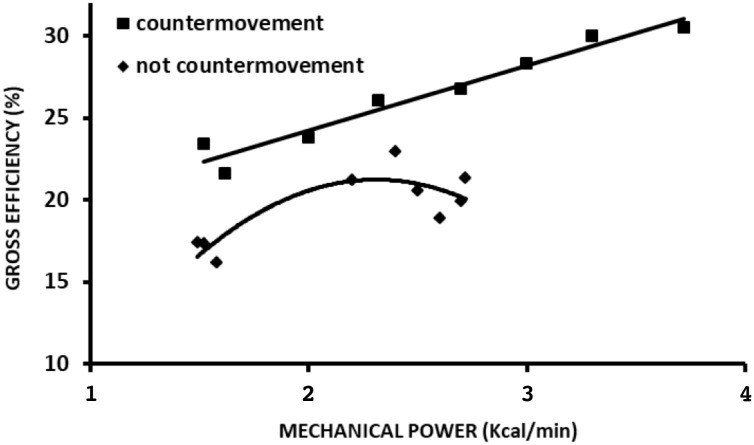
Overall efficiency during jumping in countermovement and no countermovement jumps across different mechanical power values. Data adapted from Asmussen and Bonde-Petersen (1974).

Almost a century ago, similar maximal efficiency values around 25% and at moderate speeds were observed during arm and cycle ergometer exercises (Benedict and Cathcart, 1913; Hill, 1922; Lupton and Hill, 1923; Dickinson, 1929). Margaria (1938) described that the locomotion efficiency during uphill walking was similar to that observed in an isolated muscle performing W+. Such characteristics can be observed in different modes of exercise, such as stair climbing (Lupton and Hill, 1923) (Figure 2), cycle ergometer (Dickinson, 1929; Di Prampero, 2000; Tokui and Hirakoba, 2007) (Figure 3A), and continuous vertical jumps without countermovement (Asmussen and Bonde-Petersen, 1974) (Figure 4). In these cases, *eff* is quite similar to that observed in *eff_musc_* (first approach). In addition, when the relations between load and speed are intensively manipulated (Figure 3B, Coast and Welch, 1985; Tokui and Hirakoba, 2007), the *eff_musc_* is influenced by the muscle force-velocity relationship (Hill, 1938) and fiber composition (Heglund and Cavagna, 1985; Coyle et al., 1992).

Based on perspective of transmission *eff* (second approach) it is possible accounting the mechanical work that in some cases was disregarded in muscle *eff* studies, thereby resulting in underestimated values of *eff*. Thus, it is possible to identify factors that influence transmission *eff* in an environment with different constraints. This reasoning enables us to understand *eff* in a more complex mode, not only determined by cellular physiology but also influenced by biomechanical and anatomical factors (Cavanagh and Kram, 1985a,b). Unlike the first approach, in which *eff* is predominantly influenced by *eff_musc_*; in the second approach, *eff_transmission_* plays a greater role (Figure 1B). Therefore, *eff* and *C*_metab_ sometimes do not present quasi-parabolic behavior, and even when it does, these parameters are in phase opposition (*eff* ≠ 1/*C*_metab_), and maximal *eff* values may be higher than 25–30% as well. These characteristics can be observed, for instance, in activities, such as walking and running on a level ground, (Cavagna and Kaneko, 1977, Figure 5), running downhill (Margaria, 1938, 1968), and continuous vertical jumps with countermovement (Asmussen and Bonde-Petersen, 1974, Figure 4). According to this analysis, it is possible to understand that efficiency and economy do not necessarily have an inverse and deterministic relationship, and that an economic task or activity presupposes efficiency; however, an efficient activity is not necessarily energetically economic (Minetti, 2004).

**FIGURE 5:**
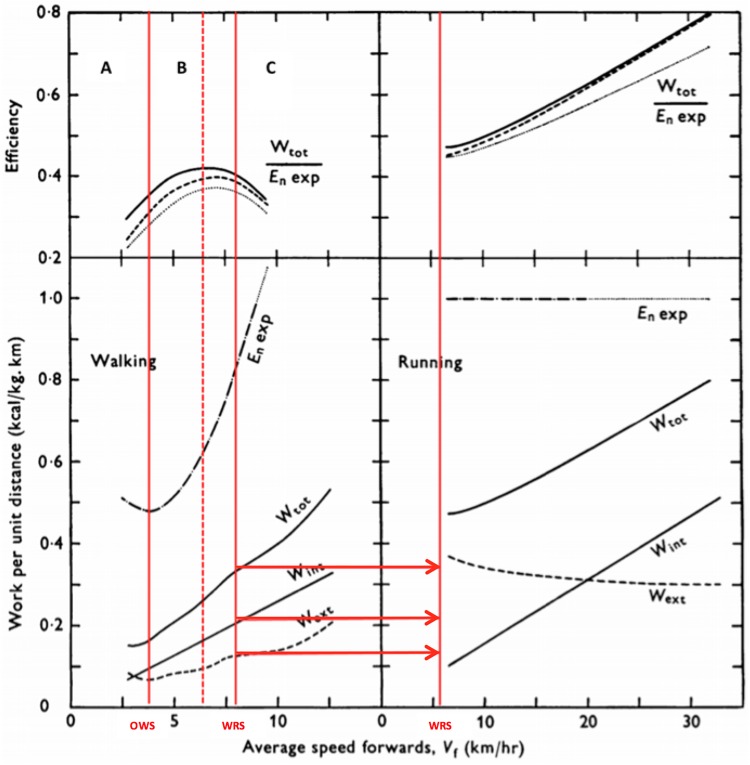
Mechanical cost (total, internal and external), metabolic cost and overall efficiency during walking and running at different velocities. Red thick line represents optimal walking speed (OWS) and transition walking-running speed (WRS) for left side (walking) and right side (running). Red dashed line represent maximal efficiency speed for walking. The letter A represents the walking speeds below OWS, the letter B represents the walking speeds between OWS and WRS, and the letter C represents walking speeds above the WRS. See further explanation for letters on text. Adapted from Cavagna and Kaneko (1977). Wext – external mechanical work; Wint – internal mechanical work; Wot – total mechanical work; En Exp – metabolic cost.

There are at least two factors that allow characterizing and dividing the activities described above between the two approaches of analyzing the economy and *eff* relation: the interplay between W+ and W- and the participation of energy-saving mechanisms. In the muscular approach, there is a predominance of concentric contractions for performance. Consequently, W+, which is more metabolic energy-consuming in nature when compared to W-, is predominantly produced (Abbott et al., 1952). Another crucial characteristic is the significant decrease or non-existence of energy-saving mechanisms, which further increases the relevance of W+ for performance. In the transmission approach, it is possible to verify similarities in the production of W+ and W-, with the concentric and eccentric contraction phases slightly different or the predominance of W- in relation to W+. We can also verify the presence of an energy-saving elastic mechanism acting with greater importance, enabling effective extra-work production to contain increases in energy expenditure. Therefore, while the first approach verified activities in which the performance is more expensive from a point of view relative, because the Wtot depended largely on the W+; in the second approach, the work produced is maximized in relation to the generated *C*_metab_.

However, some further factors interfere with the relation between *eff* and *C*_metab_, regardless of the approach analyzed. Isometric muscle contraction is an example of a condition in which there is metabolic energy consumption without production of the mechanical work (Bolstad and Ersland, 1978). The same occurs in exercise with coactivation of muscles acting antagonistically to stabilize the movement (Mian et al., 2006) without production of “useful” work. Depending on the activity (Thys et al., 1996; Pellegrini et al., 2017), the mode is performed (McMahon et al., 1987; Massaad et al., 2007) or on sample features (Mian et al., 2006). This condition may be more or less active. In these situations, *C*_metab_ will be higher and *eff* will decrease. As we will discuss ahead, in some cases, even with the existence of saving mechanisms, *eff* may be smaller than 25%.

These matters are important, because the increase or decrease in the value of *eff* is sometimes used as an argument to explain differences in *C*_metab_ between activities or locomotion situations (Thys et al., 1996). However, in some cases, this argument is not valid. For instance, comparing muscle contraction in the lower and upper limbs, or in muscles made up of different types of fibers (Heglund and Cavagna, 1985), *eff* may be the cause of differences in *C*_metab_ (*eff* of lower limb exercise higher than that of upper limb exercise, Pogliaghi et al., 2006). However, when differences lie in the amount of isometric contraction and coactivations, changes in *eff* will be a consequence rather than the cause. These examples express that *eff* and economy are not interchangeable concepts, and that regardless of approaching, extra factors may add changes in this relationship. We summarize both efficiency and economy relationship approaches as follow:

1. Corollaries of first approach (muscle perspective): (i) the efficiency and economy have quasi-parabolic behavior between different intensities; (ii) efficiency and economy have inverse and deterministic relationship; (iii) maximal efficiency value is close to 0.25 in healthy individuals and in normal environments; (iv) the major economy intensity is similar to major efficiency intensity; (v) the predominant muscle contraction is concentric; (vi) W+ is the most predominant; (vii) whole body activities with this perspective have similar characteristics to muscle isolated; (viii) other factors may change these results (e.g., isometric and co-contractions, cardiorespiratory work increase, and diseases).
2. Corollaries of second approach (transmission perspective): (i) the efficiency and economy may or not have quasi-parabolic behavior between different intensities; (ii) efficiency and economy not have inverse and deterministic relationship; (iii) maximal efficiency value is not close to 0.25 in healthy individuals and in normal environments, may be higher depending of transmission characteristics; (iv) the major economy intensity is not similar to major efficiency intensity; (v) the predominant muscle contraction is eccentric or concentric and eccentric are similar; (vi) W- is the most predominant or W+ and W- are similar; (vii) whole body activities with this perspective have not similar characteristics to muscle isolated; (viii) other factors may change these results (e.g., isometric and co-contractions, cardiorespiratory work increase, and diseases ).

In this sense, the objective of discussing the relation between *C*_metab_ and *eff* as two approaches is not to affirm the existence of two types of *eff* or to propose a new nomenclature, but only to systematize types of locomotion that present *eff* with characteristics that are similar or not to those verified for *eff_musc_* (*in situ*) and to discuss the implication of this in relation to the study of performance and physical training. The accepted difference between muscle and muscular efficiencies appear limited to us, because, in addition to the confusion caused by the similarity of these terms, it does not suit a proper classification of full-body activities through strict relations between *eff* and *C*_metab_. Energy-saving mechanisms, different interplays between W+ and W-, and other types of constraints interfere in this relation. These have been the reason for theoretical and methodological discussions, and the source of many denominations of efficiency.

Thus, based on these two efficiency approaches, is it possible to give to *eff* a performance-limiting role? Can it provide us with information about physical exercise monitoring and control? By analyzing efficiencies during walking, running, jumping, or cycling, in different restrictions, it is possible, for instance, to establish correlations between *P*_mec_ and *P*_metab_, or *C*_mec_ and *C*_metab_ (1/economy) to obtain useful information for the understanding of performance.

### Efficiency, Economy, and Power Relationship in Walking and Running

Walking and running are the main modes of human locomotion. Due to inherent complexity, it is necessary to first understand the relations between *eff* and economy at different speeds on level. The relationship between *eff*, economy, and power during walking and running are not similar, and change with speed in distinct ways. We can highlight three different patterns of *C*_metab_, *eff*, and *P*_metab_ during walking on a level ground (Cavagna and Kaneko, 1977): (A) at speeds approaching OWS, *C*_metab_ reduces, whereas *eff* increases up to values around 30%; in addition, Wext is higher than Wint, i.e., mechanical energy fluctuations of the center of body mass with respect to the environment are higher than mechanical energy fluctuations of segments with respect to the center of body mass; (B) at speeds between OWS and the walk–run transition, *C*_metab_ values increase, while *eff*, around 35%, has not yet reached its highest values. That is, the economy drops (*C*_metab_ increases), whereas the efficiency rises with increasing speed and maximal *eff* is reached only faster (40%). Contrary to situation (A), mechanical energy fluctuations here, because of the movement of limbs relative to the center of body mass (Wint), are higher than the mechanical energy fluctuations of the center of body mass with respect to the environment (Wext); (C) from the walk–run transition speed on, *C*_metab_ continues to increase, whereas *eff* decreases to values around 30%. Although *eff* and *C*_metab_ curves show quasi-parabolic behaviors in phase opposition, the walking speed at which *eff* is maximal does not correspond to the speed of lowest *C*_metab_ (OWS, Figure 5). Concomitantly, in all three situations, both *P*_mec_ and *P*_metab_ increase progressively as walking speed increases.

**FIGURE 6:**
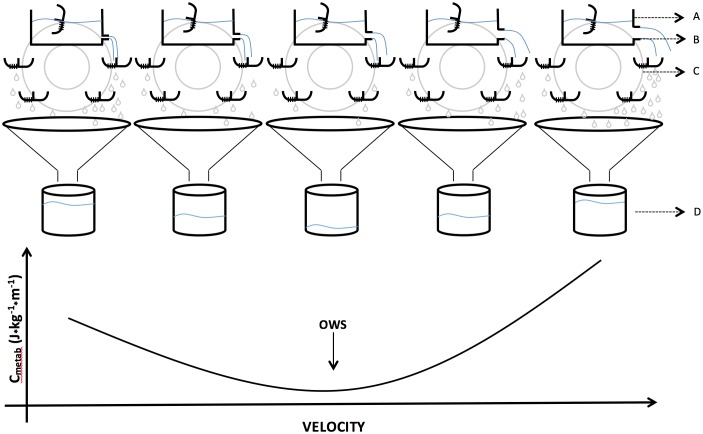
Idealization of metabolic cost (*C*_metab_) of walking based on adaptation of hydraulic model by Margaria (1976) with a watermill, representing the diameter of tube (aerobic *P*_metab_, *P*_metab_ – **B**) releasing energy/liquid (aerobic capacity tending to infinity – **A**) on the blades of a watermill (energy transduction between potential and kinetic energy – **C**). The water remaining on the blades returns to the reservoir (energy minimization) and the water that falls into the funnel represents *C*_metab_ **(D)**. Internally, the blades have two compartments (one side leaked and one not) and remain all the time facing up except for the moment when they pass through the reservoir. This model represents what might be expected into the relationship between *P*_metab_ and *C*_metab_ at progressive walking speeds under the action of an energy minimizing mechanism, that is, how the increase in power is related to the cost. The optimal walking speed (OWS) represents the velocity in which the *C*_metab_ is lower.

Unlike other settings, such as isolated skeletal muscle (Hill, 1964), cycle ergometer (Tokui and Hirakoba, 2007) and walking-climbing stairs (Lupton and Hill, 1923), it is possible to verify that higher *eff* does not explain a smaller *C*_metab_ at an OWS. The greater transduction between gravitational and kinetic energies (recovery), which characterizes the “inverted pendulum” energy-saving mechanism, largely explains the reduced value of *C*_metab_ (Figure 6) and a part of increase observed in *eff*. However, maximal *eff* values are observed at speeds higher than the OWS (Cavagna and Kaneko, 1977). Considering that *eff* can be calculated as *P*_mec_/*P*_metab_ or *C*_mec_/*C*_metab_, an increased *eff* at higher speeds is caused by a disproportionate change between the mechanical and metabolic powers values (*P*_mec_ > *P*_metab_), whereas the inversion of this increase explains the subsequent *eff* reduction (*P*_mec_ < *P*_metab_). The explanation for the disproportionate *P*_mec_ increase was attributed to the activity of the elastic mechanism at speeds above the OWS (Cavagna and Kaneko, 1977; Ishikawa et al., 2005). At speeds higher than the maximal value of *eff*, reduction in energy transduction added to an increase in respiratory cost would trigger a more considerable increase in *C*_metab_ compared with *C*_mec_, reducing *eff* (Levison and Cherniack, 1968). While recent evidences showed that the effect of this increase appears to be of little importance (Horiuchi et al., 2017), clinical studies observed that core muscle training reduced *C*_metab_ and electromyographic activities, and increased the physical performance (Finatto et al., 2018). This observation supports the knowledge of synergism between respiratory muscle activity and postural stability, as well as their associations with locomotor performance.

Energy-saving mechanisms assist to understand the behavior of the *C*_metab_ and *eff* at different walking speeds. The behavior of *C*_metab_ (U-shaped) is understood through the mechanism of the “inverted pendulum” on Wext, specifically on the transduction between Wv and Wf (Wext = Wv + Wf) generating a reduced Wext and, consequently, a reduced Wtot. This relationship changes across different speeds: at low speeds Wv > Wf and at high speeds Wv < Wf. The minimum values of *C*_metab_ coincide with Wv = Wf (Cavagna et al., 1976). However, the higher values of *eff* does not agree with major transduction energetic (recovery) (Cavagna and Kaneko, 1977). The value of *eff* continues to increase above these speeds, even with the decrease in recovery. In these velocities, the elastic mechanism is occurring (Cavagna and Kaneko, 1977) and thus, contributing to the increase in *eff*. Thus, the study of *eff* on walking needs to consider the interaction of energy transduction and elastic mechanism with the *C*_metab_ curves, otherwise divergent interpretations can be generated about the efficiency and economy relationship during walking (Donovan and Brooks, 1977).

Moreover, the transition from a “non-aerial” walk to running (bouncing) requires, in addition to changes in the pattern of neuromuscular activation, a crucial inversion as to the relationship between Wext and Wint, as Wint is greater in walking than in running, whereas Wext is lower (see horizontal red lines in Figure 5). This is in part because at speeds close to transition, the individual needs to walk at higher stride frequencies in comparison to running (Minetti et al., 1994b). At very low speeds of running, the higher Wext seems to explain the higher *C*_metab_ and *C*_mec_ values compared with walking. Despite these conditions, the *C*_metab_ of walking at high speeds may be superior to that of running (Cavagna and Kaneko, 1977; Minetti et al., 1994b). As we will discuss ahead, skipping, which is a type of locomotion that shares characteristics with walking and running, would have evolved from the need to walk at higher speeds with higher production of muscular strength and with the utilization of elastic energy (Minetti, 1998).

The relationships between running power, economy, and *eff* are different from those observed during walking. With an increased speed, there is also a progressive increase in *P*_mec_ and *P*_metab_, however, with the maintenance of *C*_metab_ and linear increase of *eff* (Cavagna and Kaneko, 1977). Maintenance of *C*_metab_ seems to be related to increased elastic mechanism, which seems to be explained by a progressive increase in stiffness (Cavagna et al., 1988). More recently, it has been verified that the increase of the push (W+) on the ground with increasing running speed improves the “elastic” rebound of the body by augmenting the role of tendons relative to muscle within muscle-tendon units (Cavagna, 2006, 2009). Currently, there are discussions around the existence of an optimal running speed in which *C*_metab_ would be minimized at a certain speed (Miller et al., 2012; Batliner et al., 2018). Probably, the body design by evolutive pressures plays a more influential role in optimal speed in walking than running. Furthermore, if it exists, this low-cost running speed could not be attributed to higher *eff*, since the *eff* continues to increase with faster speeds.

### Different Constraints Affecting Efficiency, Economy, and Power Relationship in Locomotion

The study of integrative physiology during locomotion includes the analysis of different types of constraints during walking or running. The inclined plane, carrying loads, use of accessories (crutches and poles); the effect of different environments and unusual modes of locomotion, such as skipping and jumping, are examples of constraints that can also modify the relation between economy and *eff*.

During walking on an inclined ground, there is a progressive reduction in W- production, especially from slopes above +15 to +20% (Margaria, 1938, 1968; Minetti et al., 1993), making negligible its effect on the Wtot (Margaria, 1968; Ardigò et al., 2003). There is also a progressive reduction in pendular transduction with increasing slopes (Gomeñuka et al., 2014; Dewolf et al., 2017). Therefore, the values of *eff* at different walking speeds performed from slopes above 15–20% is more as compared to the value of muscle *eff* (first approach); and the speeds of greater economy and efficiency could be closer during walking at positive slopes as compared to walking on a level ground. The reduction on performance of energy-saving mechanisms added to an increase in work against gravity could change *eff* values and relationships observed in level walking.

Walking carrying loads (extra body weight) at level and uphill incline increases the *C*_metab_, reduces the Wext, and does not change Wint, thereby reducing *eff*. However, it is unlikely that load carrying will exert independently a modification of the relationship between *C*_metab_ and *eff*, once did not provide any modification on OWS neither pendular mechanism (Bastien et al., 2005; Gomeñuka et al., 2014, 2016). Therefore, the loaded waking approaches are more likely for the muscle *eff* perspective. Increased *C*_metab_ has been attributed to greater muscle activation because of a greater requirement for postural control (Gomeñuka et al., 2016) and to decrease the coordination between the pelvic and scapular girdles (Rosa et al., 2018).

In decline walking, there is a progressive increase in W- production, with a reduction in W+ until slopes of -15 to -20% (Margaria, 1938, 1968; Minetti et al., 1993), and reduction in energy transduction (Dewolf et al., 2017). In these situations, *C*_metab_ decreases in a “U” shape, becoming more and more constant with its behavior between different walking speeds for a given negative slope (Ardigò et al., 2003). This observation represents an essential change in comparison with level walking, being similar to the behavior observed in level running. Contrary to uphill walking, the *eff* values and its relation with *C*_metab_ during downhill walking approaches the transmission *eff* perspective.

Walking with accessories (crutches and poles; NW) seems to imply changes in the locomotion economy and *eff*. The walking *C*_metab_ is superior with elbow crutches than free walking because of a higher Wtot, especially Wext, with a higher contribution of the upper limb muscles, which are energetically less efficient (Pogliaghi et al., 2006); however, they are necessary for locomotion and of greater need for isometric contractions and coactivations required for body stability. Increased mechanical work does not compensate for greater *C*_metab_, and consequently, *eff* reduced by around 12–17% (Thys et al., 1996), approximately half of the value observed in free walking (25–35%, Cavagna and Kaneko, 1977). While *C*_metab_ curve in walking with crutches has a quasi-parabolic behavior, *eff* presents linear increase with the locomotion speed. Therefore, the factors that influence both *C*_metab_ and *eff* do not affect both variables equally, since the Wtot curve also presented a quasi-parabolic behavior. Accordingly, the walking crutches approaches of transmission *eff* perspective.

In NW, the Wint of both legs and arms seem to be the factors that differ Wtot from free walking. Similar to that in walking with crutches, a higher Wtot in NW does not compensate for a higher *C*_metab_, resulting in reduced *eff* around 15 versus 19% for free walking at 4 km/h (Pellegrini et al., 2017). The pendulum mechanism is higher (67%) compared with free walking (57%) and walking with crutches (53%). Despite mechanical and *C*_metab_ differences between NW (1.7 J/kg/m) and walking with crutches (7 J/kg/m), the *eff* values are similar at the same speed and probably influenced by the same constraints.

Both walks with accessories may resemble a four-limbed biped walking (Bombieri et al., 2017); however, their mechanical and metabolic peculiarities appear to explain the differences observed regarding free walking much more than the number of limbs (Full and Tu, 1991). For instance, some studies have verified that in non-human primates, walking in biped and quadruped ways had similar values for *C*_metab_ (Taylor and Rowntree, 1973; Pontzer et al., 2014), while other studies have observed quadruped locomotion to be the most economical one (Nakatsukasa et al., 2006). Horses walking at the same speed (4 km/h) reach higher *eff* values (around 25%) with lower pendular recovery (Minetti et al., 1999). In this sense, though presenting a more significant production of mechanical work, low *eff* values can be attributed to greater muscle activation (Pellegrini et al., 2015), which raised *C*_metab_ without concomitant work generation. Thus, these modes of locomotion are examples in which relations between *C*_metab_ and *eff* differ from the *eff_musc_* approach, because it is possible the minimizing mechanisms in locomotor activities are working even when the values of efficiency are low (Cavagna et al., 1977).

The relationship between *C*_metab_ and *eff* during running at different gradients does not present a deterministic relation. Despite the progressive reduction of W- in positive slopes, the values of *C*_metab_ remains constant between different speeds, independent of the slope (Minetti et al., 1994a; Ardigò et al., 2003). This behavior may be attributed to the elastic energy released even on positive slopes (Minetti et al., 1994a), confirming the existence of an energy-saving mechanism in these conditions. Therefore, running at gradients is an example of the *eff_transmission_* approach.

Similar results were observed during loaded running. Adventure runners performed a submaximal treadmill test at 10% speed below the second ventilatory threshold, carrying loads on their backs in three different conditions (0, 7, and 15% of their individual body mass). Among the three conditions assessed, no differences were observed between the running speeds, *P*_metab_, intensity percentage of VO_2max_, heart rate, and rate of perceived exertion. However, the value of *C*_metab_ increased according to the load carried (Fagundes et al., 2017). The authors are unaware of reasons for such results, because while performance and *P*_metab_ were not impaired, *C*_metab_ increased with heavier loads. From a mechanical perspective, the authors believe that the elastic mechanism was even more optimized, because the heavier weight provided by load addition would improve the storage of elastic energy (Cavagna, 2009), thereby raising *eff* further (Cavagna and Legramandi, 2015). Conversely, the increased value of *C*_metab_ with heavier loads can be explained by a more pronounced increase of the ventilatory response verified in steady-state conditions (Ferretti et al., 2017). Thus, both running in slope and running carrying load did not change the relation between economy and *eff* observed in the level condition. The elastic mechanism probably continues influencing this relation.

Unlike walking and running, skipping is a type of locomotion rarely adopted by young people and adults; however, it is frequently and spontaneously practiced by children, and preferred by astronauts for motion in hypogravity conditions (Minetti et al., 2012; Pavei et al., 2015) together with the hopping gait (Pavei and Minetti, 2016). Recognized as a type of locomotion evolved from walking at higher speeds (Minetti, 1998), “abandoned” in the evolutionary process of hominids, skipping is also considered as an out-of-phase hopping gait (Alexander, 2004). Skipping is an interesting model of locomotion study for sharing striking characteristics with both running (aerial phase) and walking (double support). Characterized as a bouncing gait, its stride frequency values, Wint and, Wext resemble those of running and may be higher depending on speed. Nonetheless, it uses both elastic energy and inverted “pendulum” as energy-saving mechanisms. Its recovery values are similar to those of walking and reasonably high for a bouncing gait. These characteristics, coupled with the fact that its stride frequency undergoes little variation with speed, make skipping a type of locomotion that transits between walking and running, and resembles a horse’s gallop (Minetti, 1998). However, its *C*_metab_ is quite high and superior to that of walking and running in humans and horses. Despite its high *C*_metab_, its *eff* is close to the maximal values obtained during walking (40%, Minetti, 1998; Pavei and Minetti, 2016). Similar to those in level walking and running, skipping exhibits a relation between *eff* and *C*_metab_ similar to the *eff_transmission_* approach.

**FIGURE 7:**
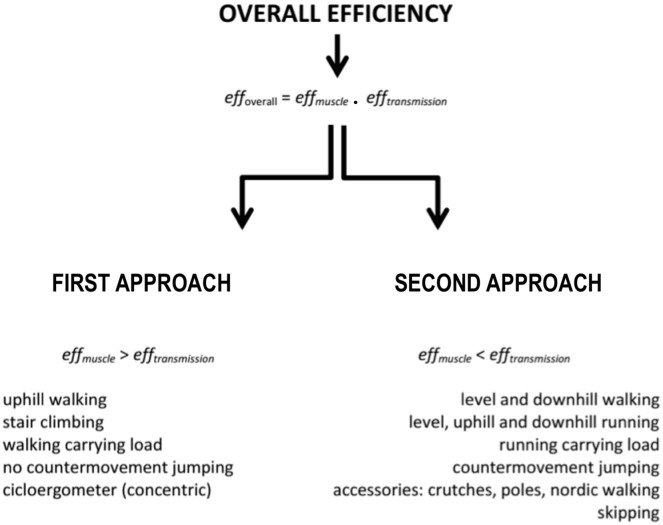
Locomotion modes and constraints organized according to the approach between efficiency (*eff*) and economy relationship. Note: *eff_overall_* – overall efficiency; *eff_muscle_* – muscle efficiency; *eff_transmission_* – transmission efficiency.

Jumping is an experimental model that differs from walking and running from the performance perspective; however, it resembles running from an energy-saving mechanism perspective. Through this type of locomotion activity, it is possible to experimentally test the effect of energy-saving mechanisms and relations between W+ and W- in both *eff* approaches. From the first perspective, it is possible to verify the effect of continuous vertical jumps without the use of countermovement, i.e., concentric contractions preceded by isometric contraction. From the second perspective, to verify the effect of continuous vertical jumps with countermovement performed by concentric contractions preceded by eccentric contractions. Analyzing the *P*_metab_ and *P*_mec_ curves of study Asmussen and Bonde-Petersen’s (1974), it was possible to plot *eff* curves for both jumping situations at different intensities. In the first approach, the *eff* curve showed a quasi-parabolic behavior with maximal values close to 25%, while in the second approach, *eff* presented linear growth with values exceeding 25%. (Figure 4). The countermovement maneuver in continuous jumps facilitated verifying the effect of the elastic mechanism on *eff*, which was similar to that observed during running; whereas in the absence of this maneuver, *eff* presented a behavior similar to that of *eff_musc_*. In addition, the energy-saving elastic mechanism and W+ and W- production play a significant role in *eff*. These results reveal that *eff* may be distinct from (countermovement jump) or similar to (squat jump) the *eff_musc_* assessed *in situ* (Figure 7).

### Efficiency, Economy, and Power Relationship in Performance and Movement Disorders

As discussed earlier, walking economy can be observed through the U-shaped analysis of *C*_metab_ at different speeds. The lowest cost value corresponds to the OWS. Interestingly, this same value corresponds to the SSWS (Margaria, 1938; Ralston, 1958). A SSWS refers to the speed chosen spontaneously by an individual for usual and consistent walking. Thus, the usual speed adopted instinctively by healthy individuals, without any type of restriction, corresponds to the most economical one.

However, several studies have shown reductions in SSWSs considering the OWS in individuals with some locomotion disorder. Regardless of age, such differences have been observed in people with hemiparesis (Schuch, 2009), lower limb amputations using prostheses (Bona, 2011), Parkinson’s disease (Monteiro et al., 2017), Machado–Joseph Disease (unpublished), patients with head trauma sequels (unpublished), heart failure and heart transplant patients (Bona et al., 2017), chronic obstructive pulmonary disease (COPD) (Sanseverino et al., 2017), and interstitial lung disease (Queiroz, 2017); and in elderly people (Gomeñuka, 2016).

Various conditions show that people with some locomotor disorder spontaneously adopt walking at less economical speeds, i.e., in the descending region of the *C*_metab_ curve. This difference may range from 40 to 90% of the OWS, depending on the type of limitation, and can be assessed through the ratio called LRI (Peyré-Tartaruga and Monteiro, 2016), which can be represented as follows:

(6) LRI=100.Self-selected walking speed/optimal walking speed

When walking more slowly, these individuals adopt lower *P*_metab_ speed; however, with higher *C*_metab_ (instead of lower *C*_metab_) and higher *P*_metab_. This “choice” may be because of mechanical (Cavagna et al., 1983) and psychophysiological reasons. When the reason is mechanical, an impact on the pendulum mechanism is noticed, thereby possibly changing its recovery, such as in individuals with prosthesis in their lower limbs (Bona, 2011). When the cause is psychophysiological, there is a type of impairment in the uptake, transport, or tissue utilization of oxygen, which reduces the muscle strength production, impairs motor control, and increases the rate of perceived exertion or dyspnea. For instance, patients with chronic heart failure have reduced LRI because they adopt a slower SSWS with higher *C*_metab_. Despite the higher energy cost, they can walk at these speeds with greater ventilatory efficiency (Figueiredo et al., 2013). The adaptations observed in these patients do not change the production of mechanical work; however, they serve to indicate the internal limitations that are acting in the determination of SSWS. Similarly, patients with Parkinson’s disease adopt slower SSWS in the same way, with higher *C*_metab_ (Maggioni et al., 2012); however, with reduced mechanical work (Dipaola et al., 2016). These observations confirm that the changes in mechanical work will not always modify *C*_metab_ in the same way. In some locomotion constraints, an increase in *C*_metab_ is caused by co-contractions, isometric contractions, and increased cardiac or respiratory work, which deteriorates the locomotion performance.

**FIGURE 8:**
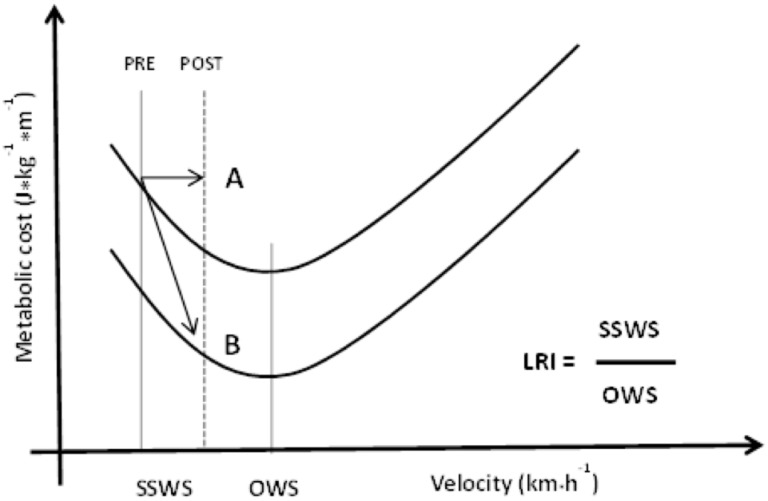
Metabolic cost at different speeds and two possible training effects for a person with locomotor disabilities. At both situations LRI increased. **A**: only increased of SSWS; **B**: increased of SSWS and decreased of metabolic cost. SSWS, self-selected walking speed; OWS, optimal walking speed; LRI, locomotor rehabilitation index.

Some intervention causes the LRI to increase, be it specifically locomotion-related or not (physical training/rehabilitation). To the best of our knowledge, no study has found changes in OWS as an effect of physical training, probably because the pendulum mechanism does not change either. Thus, the increase observed in LRI is caused by the increase in SSWS. In these situations, the LRI will be close to 100% or over. These results were observed after interventions with Parkinson’s patients (Monteiro et al., 2017), COPD patients (Coertjens et al., 2017), and old people (Gomeñuka, 2016). If the *C*_metab_ curve does not change after the intervention, the SSWS will shift to the right, and its adaptations will represent an increase in the individual’s tolerance to sustain higher intensities/speeds and at more economical speeds. Another possibility is that the *C*_metab_ curve reduces after training, thereby representing an increase in the economy at different speeds (Tam et al., 2016) and an increase in SSWS with the additional advantages in the walking economy (Figure 8).

The increase in walking economy was the result of an increase in the power output, either mechanical or metabolic. Therefore, the specific illness mechanism appears to act on *P*_mec_ and *P*_metab_, which is a critical limitation of these activities. In other words, the improvements after rehabilitation exercise interventions must permit performing displacements at *P*_metab_/intensities with more elevation. Various previous studies have confirmed an increased relationship between muscle power and usual walking velocity while walking on a plane, climbing stairs, and standing up from the chair (Cuoco et al., 2004). Therefore, one of the main objectives of the training prescription for special populations should be to specifically increase the power output (Evans, 2000). Positive variations in the walking economy based on condition A (Figure 8) may be ascribed to the changes in *P*_metab_, while in condition B, the positive adaptations are related solely to the SSWS followed by enhancement of pendular mechanism with slight changes in *P*_metab_.

In illness the reduction of *C*_metab_ during walking is related to improvement in health; while in long-distance running for highly trained athletes, the reduction in *C*_metab_ correlates with performance (Williams and Cavanagh, 1987; Tartaruga et al., 2012). Besides, these studies determined that some biomechanical parameters influence the running economy. The study by Tartaruga et al. (2012) stated that when athletes are assessed under the same metabolic conditions (same anaerobic threshold percentage) and same mechanical conditions (same speed), the stride length, stride frequency, vertical oscillation of the center of body mass, and other kinematic and neuromuscular parameters correlate with good economy, unlike in previous studies that did not have the same control.

Nonetheless, *C*_metab_ is not the only limiting parameter of performance: a high VO_2max_ (maximum *P*_metab_) and running at high %VO_2max_ are indispensable prerequisites for the analysis of the performance (Di Prampero, 1986). Therefore, both high VO_2max_ and low *C*_metab_ are important when analyzing a group of heterogeneous runners; however, in a homogeneous runners group, a higher economy seems to ensure the best performances (Tartaruga et al., 2012). Further, the value of *eff* seems to play an important role that deserves further investigation. When homogeneous runners are analyzed, while running at similar speeds and relative *P*_met_, the smaller *C*_metab_ could be explained by larger *eff*. In this case, greater efficiency could explain the higher running economy and performance. These correlations, to the best of our knowledge, have not been published and are an interesting topic for future research (Barnes and Kilding, 2015).

## Interpretations of Efficiency and Economy Relationship in Human Locomotion

The *eff_overall_* provides an exciting opportunity to investigate mechanisms and repercussions of illness, physical training, and rehabilitation intervention on functional performance in integrative physiology. In this section, we will discuss different combinations of energy expenditure as input and mechanical work as output, and their repercussions on the *eff_overall_*.

Condition 1: When the mechanical work (*C*_mec_) increases in higher proportion than the increase in *C*_metab_, it results in a greater value of *eff* (Equation 7, up and down arrows represent increase and reduction, respectively). This condition is common in bouncing gaits when the speed is increased. For example, in healthy human running, *C*_mec_ increases as a function of speed, while *C*_metab_ increases slightly because of air resistance (Cavagna and Kaneko, 1977), thereby resulting in a higher value of *eff*. This increase is because of the better functioning of elastic mechanism in muscle-tendon units. The additional storage and release of elastic energy occur during the successive steps in jumps (Pavei and Minetti, 2016), gallops (Minetti et al., 1999), and running (Cavagna et al., 1964).

(7) $$↑eff_{overall}=\frac{↑↑C_{mec}}{↑C_{metab}}$$

Condition 2: When the mechanical and *C*_metab_ reduce, the overall value of efficiency remains constant (Equation 8). One ubiquitous example of this is human walking. The pendular mechanism reduces the mechanical work required to sustain the walking movement; thus, the metabolic energy input is also reduced (Cavagna and Kaneko, 1977). Recently, Gomeñuka et al. (2014, 2016) showed that the pendular mechanism persists in the positive gradients, thereby influencing the mechanical and metabolic counterparts, and conserving the *eff_overall_*.

(8) $$≅eff_{overall}=\frac{↓C_{mec}}{↓C_{metab}}$$

Condition 3: When *C*_metab_ is increased without a corresponding elevation in *C*_mec_, the value of *eff* is reduced (Equation 9):

(9) $$↓eff_{overall}=\frac{≅C_{mec}}{↑C_{metab}}$$

These responses are caused by higher co-contraction (on running performance, Moore et al., 2014; on aging, Mian et al., 2006; Ortega and Farley, 2015) and lower mitochondrial coupling efficiency-energy conversion from oxygen uptake to adenosine-tri-phosphate production on aging (Amara et al., 2007; Conley et al., 2007). Conversely, the possible changes in the neuromuscular aspects of novice runners may account for some additional energetic optimization (e.g., Moore et al., 2012). Analyzing the data regarding *eff* and metabolic economy enables us to control these factors.

Condition 4: When *C*_metab_ is reduced without changing *C*_mec_, the value of *eff* is increased (Equation 10). Although it is seldom observed, this condition has been revealed after completing a mountain ultra-marathon, in which the running economy was enhanced with no differences in the running mechanics (Vernillo et al., 2016). It has also been observed in high-altitude trekking (115 km/day for 12 days; Tam et al., 2016) and after long cycling (170 km/day for 19 days). Unfortunately, the mechanisms underlying these observations, which states that the *eff_overall_* is increased because of improved economy, remains unclear (Vernillo et al., 2017). However, beneficial adaptations in the oxygen transport–utilization systems observed by faster VO_2_ kinetics at exercise onset (Tam et al., 2016) and changes in substrate utilization (carbohydrate to fat) are possible candidates.

(10) $$↑eff_{mec}=\frac{≅C_{mec}}{↓C_{metab}}$$

Condition 5: When *C*_metab_ is increased, and *C*_mec_ is reduced, the value of *eff* is reduced (Equation 11). In movement disorders, such as Parkinsonism because of rigidity, bradykinesia, and resting tremor, the general range of motion is reduced (Dipaola et al., 2016), thereby impacting negatively on mechanical work and *C*_metab_. Besides the LRI, *eff* seems to be a useful marker for rehabilitation in Parkinson’s disease.

(11) $$↓↓eff_{mec}=\frac{↓C_{mec}}{↑C_{metab}}$$

Condition 6: When *C*_mec_ is increased, and *C*_metab_ is reduced, the value of *eff* is increased (Equation 12). It is possible to verify this condition by customization of some conditions, such as during walking carrying loads with rubber bands on backpack (Rome et al., 2006) or using exoskeleton (Collins et al., 2015). This situation is similar to condition 1; however, the elastic energy is impacting more significantly, allowing to carry a larger mechanical load with reduction of *C*_metab_.

(12) $$↑↑eff_{mec}=\frac{↑C_{mec}}{↓C_{metab}}$$

## Conclusion

The main issues covered by this study were: (i) efficiency and economy relationship is not necessarily deterministic and inverse; (ii) it does not present the same behavior and values when analyzing different locomotor tasks; (iii) its behavior produces useful information when the influence of different restrictions, diseases, and interventions are analyzed; (iv) power can influence economy and its relationship with efficiency; thus, it is a goal to be sought during interventions. The *eff_overall_* in terrestrial locomotion is determined by *eff_musc_* (fraction of metabolic energy transformed in muscular mechanical work) and *eff_transmission_* (fraction of muscular mechanical work utilized as Wtot). Currently, the concept of *eff* is often used to determine the fraction in which metabolic energy is transformed into Wtot. Although economy and efficiency are related, they represent different energetic phenomena, and their interchangeable usage is misleading. The *eff* values close to muscle efficiency’s values (motor: 25%) indicate good *eff_transmission_* (as cycling and uphill walking); however, factors related to “machine” provide major positive work. Conversely, *eff* values higher than 25% suggest that the stretch and recoil of elastic elements in series within the muscles and tendons, provide the same or major negative work. Therefore, many combinations of mechanical and metabolic counterparts result in different possibilities of interpretation and application of economy and efficiency for different types and conditions of locomotion. In this study, we highlighted two different approaches using this relationship. This allows us to know in advance how the relationship between efficiency and economy will be, and whether there will be a deterministic relationship between the two. Further, the applications of these concepts to the integrative physiology will further improve our understanding of linkage hitherto obscure between the integrative/whole-body and cellular/molecular bioenergetics. Locomotion is a powerful model to study integrative physiology, because in the function of knowledge of its mechanical and metabolic determinants, it is possible to estimate the behavior of *eff* and economy relationship according to the locomotion type, and its characteristics and constraints, thereby allowing the intervention strategies to elaborate more adequately (e.g., physical training, rehabilitation exercise, ergogenic supplementation).

## Author Contributions

All authors listed have made a substantial, direct and intellectual contribution to the work, and approved it for publication.

## Conflict of Interest Statement

The authors declare that the research was conducted in the absence of any commercial or financial relationships that could be construed as a potential conflict of interest.

## Abbreviations

ΔH: enthalpy change
ATP: adenosine triphosphate
*C*_mec_: mechanical cost
*C*_metab_: metabolic cost
COPD: Chronic Obstructive Pulmonary Disease
*eff-*: efficiency of the negative mechanical work
*eff*_+_: efficiency of the positive mechanical work
*eff_c_*: contraction coupling efficiency
*eff_mec_*: mechanical efficiency
*eff_musc_*: muscle efficiency
*eff_overall_*: overall efficiency
*eff_p_*: phosphorylative coupling efficiency
*eff_transmission_*: transmission efficiency
f: force
LRI: Locomotor Rehabilitation Index
NW: Nordic Walking
OWS: optimal walking speed
P:O: ratio high-energy inorganic phosphates:oxygen ratio
*P*_mec_: mechanical power
*P*_metab_: metabolic power
s: space
SSWS: self-selected walking speed
VO_2_: oxygen consumption
VO_2max_: maximal oxygen consumption
W-: negative mechanical work
W+: positive mechanical work
Wel: elastic work
Wext: internal mechanical work
Wf: forward mechanical work
Wint: internal mechanical work
Wtot: total mechanical work
Wv: vertical mechanical work
